# Exome-wide search and functional annotation of genes associated in patients with severe tick-borne encephalitis in a Russian population

**DOI:** 10.1186/s12920-019-0503-x

**Published:** 2019-05-24

**Authors:** Elena V. Ignatieva, Andrey A. Yurchenko, Mikhail I. Voevoda, Nikolay S. Yudin

**Affiliations:** 1 Laboratory of Evolutionary Bioinformatics and Theoretical Genetics, The Federal Research Center Institute of Cytology and Genetics of Siberian Branch of the Russian Academy of Sciences, Novosibirsk, 630090 Russia; 2 Laboratory of Infectious Disease Genomics, The Federal Research Center Institute of Cytology and Genetics of Siberian Branch of the Russian Academy of Sciences, Novosibirsk, 630090 Russia; 3 Novosibirsk State University, Novosibirsk, 630090 Russia; 4 Research Institute of Internal and Preventive Medicine–Branch of Institute of Cytology and Genetics, Siberian Branch of Russian Academy of Sciences, Novosibirsk, 630004 Russia

**Keywords:** Tick-borne encephalitis, *Flavivirus*, Whole-exome sequencing, Candidate genes, Genetic predisposition, Biological pathways, Network, PPIs

## Abstract

**Background:**

Tick-borne encephalitis (TBE) is a viral infectious disease caused by tick-borne encephalitis virus (TBEV). TBEV infection is responsible for a variety of clinical manifestations ranging from mild fever to severe neurological illness. Genetic factors involved in the host response to TBEV that may potentially play a role in the severity of the disease are still poorly understood. In this study, using whole-exome sequencing, we aimed to identify genetic variants and genes associated with severe forms of TBE as well as biological pathways through which the identified variants may influence the severity of the disease.

**Results:**

Whole-exome sequencing data analysis was performed on 22 Russian patients with severe forms of TBE and 17 Russian individuals from the control group. We identified 2407 candidate genes harboring rare, potentially pathogenic variants in exomes of patients with TBE and not containing any rare, potentially pathogenic variants in exomes of individuals from the control group. According to DAVID tool, this set of 2407 genes was enriched with genes involved in extracellular matrix proteoglycans pathway and genes encoding proteins located at the cell periphery. A total of 154 genes/proteins from these functional groups have been shown to be involved in protein-protein interactions (PPIs) with the known candidate genes/proteins extracted from TBEVHostDB database. By ranking these genes according to the number of rare harmful minor alleles, we identified two genes (*MSR1* and *LMO7),* harboring five minor alleles, and three genes (*FLNA, PALLD, PKD1*) harboring four minor alleles.

When considering genes harboring genetic variants associated with severe forms of TBE at the suggestive *P*-value < 0.01, 46 genes containing harmful variants were identified. Out of these 46 genes, eight (*MAP4, WDFY4, ACTRT2, KLHL25, MAP2K3, MBD1, OR10J1,* and *OR2T34)* were additionally found among genes containing rare pathogenic variants identified in patients with TBE; and five genes (*WDFY4****,*** *ALK, MAP4, BNIPL, EPPK1*) were found to encode proteins that are involved in PPIs with proteins encoded by genes from TBEVHostDB. Three genes out of five (*MAP4, EPPK1, ALK*) were found to encode proteins located at cell periphery.

**Conclusions:**

Whole-exome sequencing followed by systems biology approach enabled to identify eight candidate genes (*MAP4, WDFY4, ACTRT2, KLHL25, MAP2K3, MBD1, OR10J1,* and *OR2T34*) that can potentially determine predisposition to severe forms of TBE. Analyses of the genetic risk factors for severe forms of TBE revealed a significant enrichment with genes controlling extracellular matrix proteoglycans pathway as well as genes encoding components of cell periphery.

**Electronic supplementary material:**

The online version of this article (10.1186/s12920-019-0503-x) contains supplementary material, which is available to authorized users.

## Background

Tick-borne encephalitis is a disease caused by tick-borne encephalitis virus (TBEV), which is a neurotropic, positive-sense RNA virus. Along with other important human pathogens, such as Zika virus, dengue virus, West Nile virus, and Japanese encephalitis virus, TBEV belongs to the genus *Flavivirus*, family *Flaviviridae* [1]. TBEV is endemic in an area ranging from northern China and Japan, through far-eastern Russia to Europe [2]. In these regions, TBEV occurs in forest and forest-steppe zones [3, 4].

In 2017, the incidence of tick-borne encephalitis in European countries was as follows: (1) Slovakia − 75 cases; (2) Poland - 267 cases; (3) Germany − 485 cases; (4) Czech Republic – 565 cases; (5) Austria – 89 cases [5]. According to statistics provided by the Russian Research Anti-Plague Institute “Microbe” (Saratov) of the Federal Service for Supervision of Consumer Protection of the Russian Federation (Rospotrebnadzor) [6], 509.262 people sought medical care because of tick bites in 2017, which is 6% higher than in 2016. In total, 1.943 cases of tick-borne encephalitis were registered in the Russian Federation in 2017 (of them 41 cases were reported in Moscow and 135 - in Novosibirsk region) and 28 people from 14 regions of the Russian Federation have died from tick-borne infections [7, 8].

TBEV infection can lead to a variety of clinical manifestations ranging from mild fever to severe neurological illness (meningitis, meningoencephalitis, meningoencephalomyelitis) [9, 10].

Delayed immune response of specific IgG and age above 61 are significant prognostic factors for the severe course of the disease [11]. A positive correlation between age and severity of TBE may be due to thymus involution and impaired immunity in elderly people [12]. The severity of the disease also may be determined by both genetic susceptibility of the host and genetic factors related to the virus subtypes.

Three genetically distinct subtypes of viruses within a single TBE virus serocomplex cause TBE: Far-Eastern subtype, Siberian subtype, and European subtype. Each of these subtypes cause clinically distinct diseases with varying degrees of severity [13, 14]. TBEV of European subtype generally causes a biphasic disease, occasionally resulting in neurological disorders, but with low case fatality rate (according to [15], it is 1–2%). In contrast, infection with the Far-Eastern subtype of TBEV is more frequently associated with severe neurological disorders, relatively high case fatality rate (according to [15], it is 20–40%) and increased propensity for neurological sequelae in survivors. The Siberian subtype of TBEV is intermediate in disease severity (according to [16], a mortality rate is 2–3%), but has been associated with chronic infection [12, 14].

The neuropathogenesis of TBEV is determined by the capacity of the viral particle to enter the central nervous system after peripheral inoculation and its ability to replicate and cause damage within the central nervous system [15]. After the bite of an infected tick, the virus usually replicates in the dermal cells at the site of the tick bite [17]. Later the TBE virus reaches regional lymph nodes via lymph capillaries and spreads via the lymphatic system [17]. A few days later, TBE virus reaches the bloodstream [15, 17–19]. Then, invasion of susceptible organs or tissues (in particular, reticuloendothelial system) occurs. After this stage, the virus may cross the blood-brain barrier and invade the central nervous system, where it can cause profound destruction of nerve cells [2]. It may also enter the brain directly via the fila olfactoria after infecting the neuroepithelial cells of the nasal mucous membrane [17]. The most severe forms of TBE are characterized by damage of neurons in different parts of the brain and spinal cord [9]. Generally, central nervous system pathology is the consequence of viral infection of corresponding cells and the resulting neuroinflammatory responses [19].

For decades, tick-borne encephalitis caused by TBEV infection has been a serious danger to public health [12, 20, 21]. Today, four licensed vaccines are available for the prevention of TBE, two in Europe and two in Russia [14]. Vaccines licensed for use in Europe include an Austrian vaccine (FSME–Immun®; also known as TicoVac® in all Baltic and Scandinavian countries; Pfizer formerly Baxter) and a German vaccine (Encepur®; formerly Novartis, since 2015 GSK vaccine) [12]. Two vaccines based on TBEV Far-Eastern strains are currently licensed for use in Russia Encevir® (Microgen, Tomsk, Russia) and IPVE (Institute of Polyomyelitis and Viral Encephalitis, Moscow, Russia) [14, 22]. Published studies have shown that European vaccines are cross-protective in rodent models and elicit cross-reactive neutralizing antibody responses in humans [14, 22]. A number of countries including Russia, Austria, Germany, Finland, Hungary, Latvia, Slovenia, Switzerland, and Italy have government vaccination programs [23]. In Russia, vaccination against TBE is recommended for all age groups and is available for anyone who lives in endemic areas or plans extensive outdoor activities in such areas. According to the Federal Service for Supervision of Consumer Rights Protection of the Russian Federation (Rospotrebnadzor) [6], 2.7 million Russians were vaccinated against TBEV in 2017 [7].

Despite the fact that currently there is a human TBEV vaccine available, there is no specific treatment once infected [5, 24]. There is reliable evidence that host genetic factors may contribute to susceptibility or resistance to flaviviruses [25–28].

Studies performed in mice demonstrated the effect of host genetics on the course of TBE. It was observed that mice of three different strains showed different severities of the TBEV. After subcutaneous inoculation of TBE virus, BALB/c mice showed medium susceptibility to the infection, STS mice were resistant, and CcS-11 mice (recombinant congenic strain of mice derived from the background strain BALB/c and the donor strain STS) were highly susceptible [29]. Using F2 hybrids between BALB/c and CcS-11 mice, a follow-up study was performed to determine the location of genes responsible for susceptibility to TBEV infection. Linkage analysis identified a novel suggestive locus on chromosome 7. This locus contained nine candidate genes (*Cd33, Klk1b22, Siglece, Klk1b16, Fut2, Grwd1, Abcc6, Otog,* and *Mkrn3*), which may be a focus of future studies not only in mice, but also in humans [30].

A number of knockout mouse studies have addressed the roles of relevant genes (*MAVS, TNF, IL10, CD8A, IFNAR, TIA1,* and *TIAL1*) in the host response to TBEV infection as well as their impact on the disease severity. Knockout of these genes led to (1) increased mortality rates (MAVS, TNF and IL10 KO mice) [31, 32]; (2) delayed appearance of neurological signs of disease (CD8A KO mice) [33]; (3) increased TBEV titers and RNA replication in astrocytes (IFNAR KO mice) [34]; (4) affected (increased or decreased) TBEV extracellular infectivity (TIA1 and TIAL1 KO mice) [35].

In humans, host genetic factors that control immune response to TBEV and thus may determine the severity of the TBEV infection are not well understood. Only few studies have been conducted on the genetic predisposition to severe forms of TBE in humans [36–45]. Studies performed on the Russian population have shown that five SNPs in *OAS2* and *OAS3* genes, as well as two SNPs in *IFNL3/IL28B* gene and SNPs in *TLR3, CD209*, *IL10, MMP9*, *ABCB9*, and *COL22A1* genes were associated with predisposition to severe forms of TBE [36–41]. Polymorphisms in the chemokine receptor 5 (*CCR5*) and toll-like receptor 3 (*TLR3*) genes were found to be risk factors for clinical tick-borne encephalitis in the Lithuanian population [42–44]. In addition, it was reported that intronic polymorphisms in *IFNL4* (rs12979860) and *ARID1B* (rs287886) genes were associated with IFNL3 and IL-10 concentrations in cerebrospinal fluid of TBE patients in the Polish population [45].

In our recent report [46], we have presented a catalog of human genes that can be involved in response to TBEV infection (TBEVHostDB, http://icg.nsc.ru/TBEVHostDB/). TBEVHostDB includes data on 140 candidate genes identified based on manually collected information from a number of research papers. Genes were classified into five functional categories according to the type of evidence that confirms their involvement in the host response: (1) genes encoding proteins involved in direct physical interactions with TBE viral particle, TBEV proteins or RNA; (2) genes encoding mRNAs (or proteins) that were up- or down-regulated in response to TBEV infection; (3) genes that had allelic variant associated with susceptibility or resistance to TBEV infection; (4) genes encoding proteins required for the inhibitory effect of other proteins against TBEV or proteins that attenuated antiviral activity of other proteins; (5) genes that have been knocked out in mice and, as a consequence, mortality rates or other clinical manifestations of the disease were increased [46].

As costs for genome sequencing further decrease and technologies become more advanced, genome- and exome-wide association studies (GWAS or EWAS) are becoming an increasingly widely used tool for identifying genetic risk factors for various diseases [47, 48]. Recently, the results of the first pilot study based on whole-exome sequencing of DNA samples from six Novosibirsk TBE patients with severe forms of TBE and seven individuals from the control group, have been published [40]. In this pilot study, we applied whole-exome sequencing combined with the candidate gene approach, and, as a result, we identified nonsynonymous SNP (rs17576) in *MMP9* gene being a new locus associated with predisposition to TBE in the Russian population.

The goals of this study were (1) to perform whole-exome sequencing of DNA from blood samples of Russian patients with severe forms of TBE; (2) to identify genetic variants associated with severe forms of TBE; and (3) to determine biological pathways through which the identified variants may influence the severity of the disease.

## Methods

### Subjects

Blood samples were collected from 16 unrelated symptomatic patients with severe (meningo-encephalitic or meningo-encephalo-poliomyelitic) forms of TBE who were treated in specialized infectious hospitals in Novosibirsk (Russia) between 2002 and 2007 years. Only patients with clinically confirmed TBE diagnosis according to standard criteria [13] based on clinical symptoms, seasonality, evidence of tick bite, cerebrospinal fluid examination and positive immunological diagnosis were used in our study. All TBE patients gave informed consent for participation in the study. Only those patients who self-reported that they had not previously received a TBE virus vaccination or specific immunoglobulin after a tick bite were included in the study***.*** The research was approved by the Bioethics Committee of the Federal Research Center Institute of Cytology and Genetics of Siberian Branch of the Russian Academy of Sciences. We also used the whole-exome sequencing data of six patients diagnosed with severe forms of TBE and 17 control subjects that were described previously [40, 49]. This control cohort consisted of randomly selected healthy individuals in the same Novosibirsk districts. No information about previous TBE virus infection was available. All blood samples were taken from Caucasians, mainly Russian individuals of both sexes. The group of patients with severe forms of TBE (18 patients had meningo-encephalitic form and 4 patients had meningo-encephalo-poliomyelitic form of TBE) included 13 men and 9 women aged 42.3 + 5.7 and 53.8 + 4.9 years respectively. This group of patients contained approximately 20% of the total number of patients with severe forms of TBE registered between 2002 and 2007 years. Control samples were collected from 9 men and 8 women aged 39.4 + 7.4 and 38.3 + 7.3 years respectively. DNA was extracted from the whole blood by phenol and chloroform deproteinization [50]. DNA was extracted soon after collection.

### Exome sequencing and data analysis

The preparation of libraries and exome enrichment were performed with an Agilent SureSelect Human All exon V5 Kit (Agilent Technologies, USA). Sequencing was carried out by the BGI (Hong Kong) on a HiSeq Illumina 4000 platform (Illumina Inc., San Diego, CA, USA) according to the manufacturer’s protocols. Raw sequencing reads (150 bp, paired-ends) were checked with the FastQC program (version 0.11.5) [51]. The remains of adapters, reads with unknown nucleotides, and low quality nucleotides were removed using the Trimmomatic program [52]. The reads that passed the quality assessment were aligned to the reference Hg19 genome with the BWA program [53], and the resulting BAM files were sorted using the Samtools program [54]. Genetic variants were identified in the BAM files using the GATK HaplotypeCaller program [55]. The phred-scaled read mapping quality threshold was set to 50 and the nucleotide phred-scaled quality was set to 30. The obtained genetic variants were filtered by location (target sequences of the Agilent SureSelect Human All exon V5 Kit) and quality (minimum phred-scale quality> 50, genotype quality> 20, ≤ four samples with missed variants). As we combined our whole-exome sequencing data with the full exome data obtained previously [40, 49], at this step the search for variants was carried out simultaneously for all exomes (22 Russian patients with severe forms of TBE and 17 Russian individuals from the control cohort).

To establish a functional class of the variant (synonymous substitutions, nonsense and missense mutations) and to identify pathogenic (harmful) variants we used ANNOVAR software [56]. The variant was considered harmful, if it was pathogenic or probably pathogenic according to SIFT [57], or PolyPhen2 hvar or Div [58]. To identify variants that are not common (MAF < 0.05) in the non-Finnish European (NFE) populations the ExAC database [59] was used.

Identification of genetic variants associated with severe forms of TBE in 39 exomes of Russian individuals (22 patients with TBE and 17 individuals from the control cohort) was performed using PLINK software with 1 million permutations (−-assoc fisher-midp mperm = 1,000,000). Array-wide significance was defined as *P* < 3 * 10^− 7^ [60], suggestive significance was defined as *P* < 1 * 10^− 2^ due to the low sample size used in the work.

### GO category and pathway analysis

The identification of GO Categories and canonical pathways enriched in gene sets was performed using a web-based functional annotation tool known as the DAVID (Database for Annotation, Visualization and Integrated Discovery) tool [61] against the ‘whole genome’ background. The tool allows detection of enriched terms from Gene Ontology and biological pathways or models for a variety of biological processes presented in the REACTOME, KEGG, and BIOCARTA. The benefits of using the ontological and pathway analyses for functional annotation of genes harboring SNPs, associated with various traits, have been confirmed in numerous publications [62–66].

### Indexing genes according to the number of the harmful minor alleles

The value of the index N, corresponding to the number of rare harmful minor alleles that were found in exomes of patients with TBE, was calculated as follows. If the gene J contained harmful minor allelic variants in M loci, and that minor variants were not common in NFE populations (MAF < 0.05), then N*j* was computed as a sum of K*i*, where:

1. *i* = 1, ..,M;
2. K*i* was equal to the total number of the minor alleles found in the loci I in the set of exomes of TBE patients. For the patient possessing certain minor allele in loci I, the occurrence of this minor allele was equal to 1, if the patient had heterozygous genotype. The occurrence was equal to 2, if the patient possessed both harmful alleles in loci I (homozygous genotype).

### Identification of PPIs between genes/proteins from the ECM proteoglycans - cell periphery dataset and genes/proteins from the TBEVHostDB

To identify pairwise physical interactions between genes/proteins we employed the GeneMANIA Cytoscape plugin [67]. The combined list of genes that included 879 genes/proteins from the ECM proteoglycans - cell periphery set and 140 genes/proteins from the TBEVHostDB [46] was used for query to GeneMANIA. Thus, the initial PPI network was reconstructed. In addition to interactions between genes/proteins from the ECM proteoglycans - cell periphery set and genes/proteins from the TBEVHostDB this network involved interactions involving either both genes from the *ECM proteoglycans - cell periphery* set or both genes from TBEVHostDB. To identify a subset of genes/proteins from the *ECM proteoglycans - cell periphery* set that had physical interactions with genes/proteins from the TBEVHostDB only, we used the following two Cytoscape options: (1) *First neighbors of selected nodes;* and (2) *New network from selection.* Thus, the PPI network involving only interactions between genes/proteins from the *ECM proteoglycans - cell periphery* set (as a first interactant) and genes/proteins from the TBEVHostDB (as a second interactant) was reconstructed. Data on physical interactions were extracted in a tab-delimited format, and, if more than one edge connected two nodes in this network, the edge with the maximal weight was selected for further consideration. Then data again were imported into Cytoscape. For each gene/protein the following additional data were imported into Cytoscape as attributes of nodes and used to arrange the visualization style: (1) whether the gene/protein is present in the TBEVHostDB; (2) the index N, which was described above.

## Results

### Exome-wide search for genes associated with severe forms of tick-borne encephalitis

#### Identification of genetic variants in the exomes of the TBE patients and in the control samples

We sequenced the exomes from 16 Novosibirsk TBE patients with severe CNS disease. As a result, 18.6–29.8 million “raw” paired-end reads per sample were obtained. After removal of low-quality reads, clean reads, constituting from 84.1 to 85.9% of the total, were retained for further analysis. The median coverage for different samples ranged from 41X to 63X and the mean coverage ranged from 48X to 71X. This depth of coverage allows accurate base calling of single nucleotide variants (SNVs) [68]. The percentage of concordantly mapped paired-end reads ranged from 95.7 to 99.6%. As a result, after combining these data with the sequencing data obtained previously [40, 49], 128,739 high-quality SNVs and 12,562 short insertion/deletion variants were identified in 39 exomes (22 patients with TBE and 17 control healthy individuals in total).

#### Detection of harmful genetic variants that are not common in non-Finnish European populations and finding genes harboring these variants

The high-quality genetic variants identified at the previous step were annotated with ANNOVAR software and filtered with the following criteria: (1) the variant is not common (MAF < 0.05) in NFE populations according to the ExAC database; (2) the variant is pathogenic or probably pathogenic according to the PolyPhen2 hvar or Div or SIFT databases. In total, there were 6141 such variants in the patient’s exome sample (17 indels and 6124 SNVs) and 4869 variants in the control exome sample (11 indels and 4858 SNVs).

**Fig. 1:**
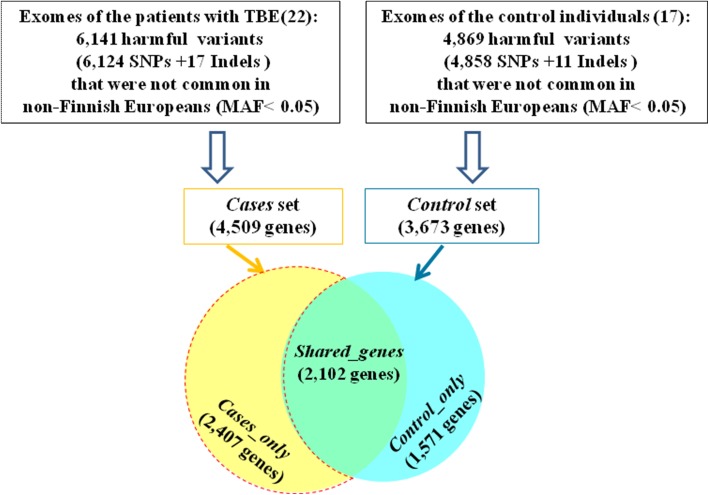
Harmful variants that were not common in non-Finnish Europeans (MAF < 0.05) identified in exomes of patients with TBE and control individuals (up) and Venn diagram representing the numbers of genes harboring these harmful variants (down). The *cases_only* gene set is outlined by red dotted line

It was estimated that rare pathogenic variants identified in exomes of patients with TBE were located in 4509 genes (this gene set is designated below as *cases* set) (Table 1, Additional file 1: Table S1). Pathogenic variants found in exomes of the control individuals were located in 3673 genes (designated below as *control* set, (Additional file 1: Table S1)). These two gene sets (*cases* set and *control* set) had 2102 shared genes (designated below as *shared* set) (Fig. 1). Besides, 1571 genes were unique for the *control* set and 2407 genes were unique for the *cases* set. This set, comprising 2407 unique genes (denoted in the Fig. 1 by the red dotted line) will be designated below as *cases_only set*. Using the procedure described above (see Methods section), the following values of index N were calculated for these genes (Additional file 1: Table S2).

**Table 1:** Sets of genes used in analysis

Gene set	Description	Number of genes / proteins
*Cases*	Genes harboring pathogenic variants found in exomes of patients with TBE, that were not common in non-Finnish Europeans (MAF < 0.05)	4509
*Control*	Genes harboring pathogenic variants found in exomes of individuals from the control cohort, that were not common in non-Finnish Europeans (MAF < 0.05)	3684
*Cases_only*	Genes harboring rare pathogenic variants in exomes of patients with TBE only and not containing any rare pathogenic variants in exomes of individuals from the control cohort.	2407
*ECM proteoglycans - cell periphery* set	Genes from the *cases_only* set that, according to DAVID tool, are annotated by at least one of the following four enriched GO terms: (1) *cell periphery; (2) plasma membrane; (3) plasma membrane part; (4) integral component of plasma membrane;* or belonging to *ECM proteoglycans pathway*	749
*TBEVHostDB*	Genes that are probably involved in response to TBEV infection (http://icg.nsc.ru/TBEVHostDB/)	140
*Associated*	Genes harboring genetic variants associated with severe forms of TBE at *P*-value < 0.01. The search of associated variants was performed based on whole-exome sequencing of 22 patients with TBE and 17 control individuals with PLINK software.	667
*Associated_possibly damaging*	Genes from the *associated* set that harbored only potentially damaging genetic variants. The following types of variants were considered to be potentially damaging: all nonsynonymous, frameshift and stopgain variants, as well as variants annotated by PolyPhen2 or SIFT databases as probably damaging or possibly damaging.	132
*Associated_harmful*	Genes from the *associated* set that harbored harmful genetic variants. The following types of variants were considered to be harmful: (1) variants annotated by PolyPhen2 or SIFT databases as probably damaging or possibly damaging, as well as (2) one additional stopgain variant.	46

#### SNVs and genes associated with severe central nervous system disease

The genotypes from two Russian sets (22 patients with meningo-encephalitic or meningo-encephalo-poliomyelitic forms of TBE and 17 healthy individuals from the control group) were analyzed with PLINK software.

**Fig. 2:**
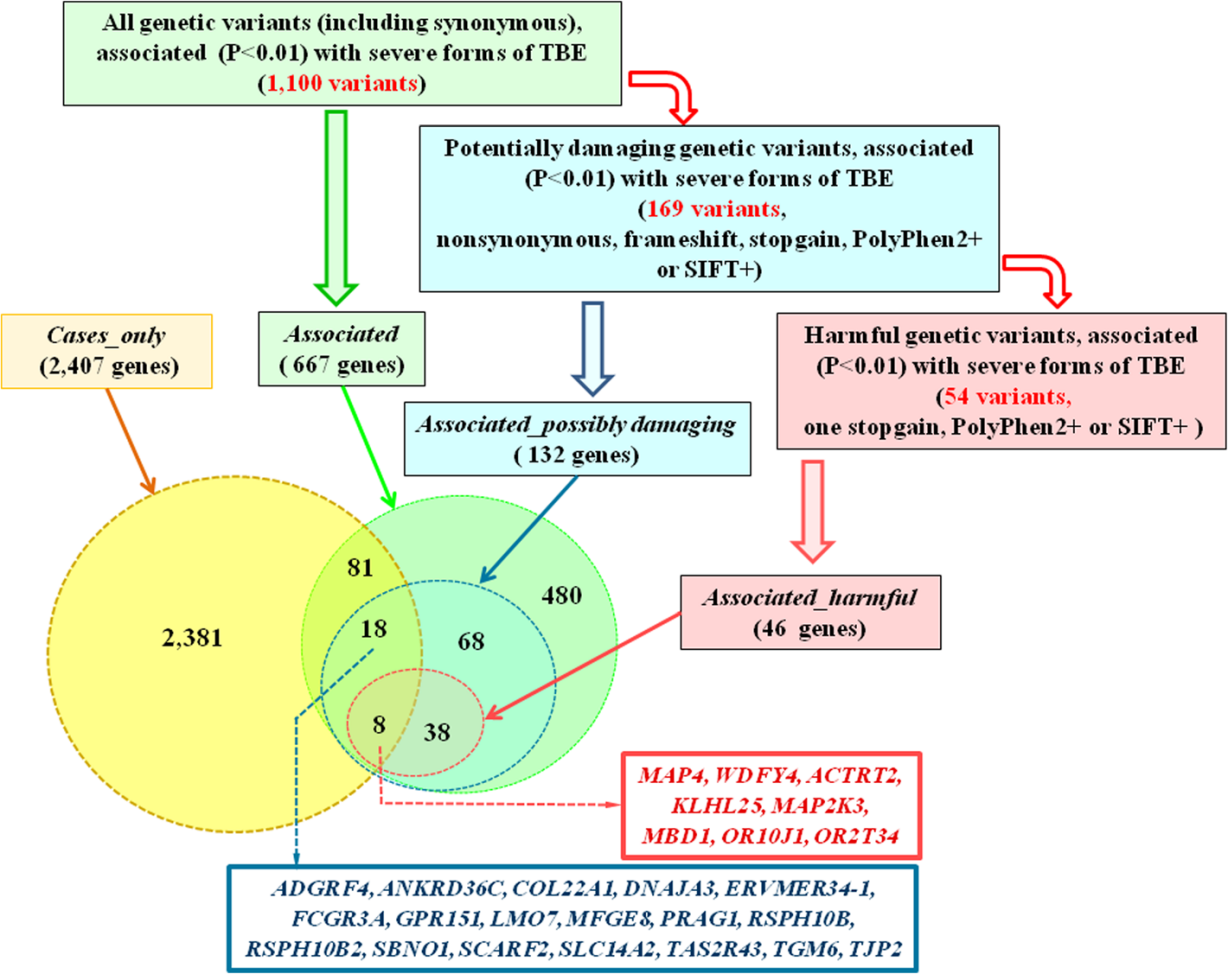
Genetic variants associated with severe forms of TBE (up) and Venn diagram representing the numbers of genes harboring these genetic variants and genes from the c*ases_only* set, which was described above (down)

We were unable to identify genetic variants with *P* values reaching array-wide significance (< 3 * 10^− 7^). Perhaps this was due to low sample sizes and complex genomic background of the tick-borne encephalitis. That is why we chose genetic variants with *P*-values satisfying suggestive significance level of 0.01 and proposed that genes harboring such variants may be involved in the pathogenesis of TBEV infection. We found 1100 genetic variants associated with severe forms of TBE at P value less than 0.01 (Fig. 2, Additional file 1: Table S3). Among them 1058 were known polymorphisms that were annotated in dbSNP, and the other 42 were newly identified SNVs. About 17% of variants were synonymous, 19% were nonsynonymous, and 0.8% were stopgain or frameshift. These 1100 genetic variants were located within (or in vicinity of) 677 genes (designated below as *associated* set (Fig. 2, Table 1, Additional file 1: Table S4)). Sixteen percent of these 677 genes (107 genes) were also contained in the *cases_only* set (Fig. 2).

Of particular interest were genes harboring pathogenic variants that may be harmful for the protein function. Thus, we created two sets of genetic variants: (1) the set of potentially damaging genetic variants that included all nonsynonymous, frameshift and stopgain variants, as well as variants annotated by PolyPhen2 or SIFT databases as possibly or probably damaging; (2) the set of harmful genetic variants that included variants annotated by PolyPhen2 or SIFT databases as possibly or probably damaging, as well as one additional stopgain variant. In what follows, the corresponding sets of genes harboring these variants will be designated as *associated_possibly damaging* set (including 132 genes) and *associated_harmful* set (including 46 genes) (Fig. 2, Table 1 and Additional file 1: Table S4).

The comparison of genes from the *associated_possibly damaging* (132 genes) set and *cases_only* set identified 26 shared genes (Fig. 2). Eight of these 26 genes (*ACTRT2, KLHL25, MAP2K3, MAP4, MBD1, OR10J1, OR2T34,* and *WDFY4*) were also shared between the *associated_harmful* (46 genes) and *cases_only* sets. The genetic variants found in these eight genes are presented in Table 2. For most of them, we found one harmful variant that is not common in non-Finnish Europeans and one harmful variant associated with severe forms of TBE. Two genetic variants associated with severe forms of TBE were found in *OR10J1* and *MAP4*.

**Table 2:** Genetic variants found in eight genes shared by the *cases_only* (2407 genes) set and the *associated_harmful* (46 genes) set

Gene	Harmful genetic variants found in patients with TBE, that are not common in non-Finnish European populations						Harmful genetic variants associated with severe forms of TBE				
	SNP ID or SNV position^a^	Exonic function	SIFT	Polyphen2 HDIV	Polyphen2 HVAR	N ^b^	SNP ID	*P*-value	Exonic function	SIFT	Polyphen2 HDIV
*MAP4* ^c^	rs150907099	nonsyn	D	D	D	1	rs11711953	0.001927	nonsyn	D	D
							rs2230169	0.005399	nonsyn	D	D
*WDFY4*	chr10:50,034,917	nonsyn	D	D	D	1	rs41283283	0.003018	nonsyn	T	D
*ACTRT2*	rs137950147	nonsyn	T	D	P	1	rs3795263	0.009637	nonsyn	D	D
*KLHL25*	rs779814173	nonsyn	D	B	B	1	rs35582838	0.009274	nonsyn	D	B
*MAP2K3*	rs141390631	nonsyn	D	P	D	1	rs74575904	0.002442	nonsyn	D	D
*MBD1*	rs183864846	nonsyn	D	B	B	1	rs125555	0.009685	nonsyn	D	P
*OR10J1*	rs35634161	nonsyn	D	D	D	2	rs12048482	0.003306	nonsyn	D	D
							rs12409540	0.005492	stopgain	.	.
*OR2T34*	rs202166093	nonsyn	D	D	D	1	rs199863869	0.001284	nonsyn	D	B

^a^SNV genomic position was determined according to the GRCh37/hg19 assembly

^b^N - the occurrence of the rare harmful minor alleles harbored by certain gene in exomes of TBE patients (see Methods section)

^c^*MAP4* has PPI with one gene (*IL7R*) from the TBEVHostDB

The genetic variants found in the rest 18 genes indicated as shared in Fig. 2 are presented in Additional file 1: Table S5. For all of them we identified at least one harmful variant that is not common in non-Finnish Europeans and one nonsynonymous or frameshift variant associated with severe forms of TBE.

### Functional annotation of genes associated with severe forms of tick-borne encephalitis

#### Identification of genes that have evidence in the TBEVHostDB

Next, we examined if the *cases_only* set *and associated* sets contained genes that are already known to be associated with susceptibility to TBE disease. For this purpose, we used TBEVHostDB - a catalog of human genes that are potentially involved in response to TBEV infection [46].

Expecting the *cases_only* set (2407 genes), we found that, according to the TBEVHostDB [46], nineteen genes may be involved in response to TBEV infection. Of these nineteen genes (1) one gene (*TLR3*) had allelic variant associated with susceptibility or resistance to TBEV infection; (2) six genes (*TYK2, FAM184A, PHC2, TJP2, SCRIB,* and *ITGB3*) encoded proteins that had direct physical interactions with TBEV virion, TBEV proteins or TBEV RNA; and (3) 12 genes (*A2M, HMOX1, ICAM3, IFIH1, IL12B, HSPA1A, IL2RA, IFIT1, CXCL12, ICAM1, LTF,* and *GFAP*) encoded mRNAs (or proteins) that were up- or down-regulated in response to TBEV infection (Table 3) .

**Table 3:** Genes from the *cases_only* set and the *associated* set, which are characterized in the TBEVHostDB

Gene	Functional group of genes from the TBEVHostDB ^a^	Reference from the TBEVHostDB	Number of polymorphisms per gene ^b^
*Cases_only* set of genes			
*TLR3*	Allelic variant	[38, 44]	3
*TYK2*	Physical interaction	[69]	1
*FAM184A*	Physical interaction	[69]	1
*PHC2*	Physical interaction	[69]	1
*TJP2*	Physical interaction	[70]	1
*SCRIB*	Physical interaction	[71]	1
*ITGB3*	Physical interaction	[72]	1
*A2M*	Up- or down-regulated	[73]	2
*HMOX1*	Up- or down-regulated	[74]	1
*ICAM3*	Up- or down-regulated	[75]	1
*IFIH1*	Up- or down-regulated	[19]	1
*IL12B*	Up- or down-regulated	[76]	2
*HSPA1A*	Up- or down-regulated	[77]	1
*IL2RA*	Up- or down-regulated	[78]	1
*IFIT1*	Up- or down-regulated	[79]	1
*CXCL12*	Up- or down-regulated	[17]	1
*ICAM1*	Up- or down-regulated	[78]	1
*LTF*	Up- or down-regulated	[80]	2
*GFAP*	Up- or down-regulated	[81]	1
*Associated* set of genes			
*TJP2*	Physical interaction	[70]	1
*ATF6*	Up- or down-regulated	[77]	1
*ARID1B*	Allelic variant	[45]	1

^a^Allelic variant, Allelic variant in this gene was associated with susceptibility or resistance to TBEV infection; Physical interaction, Genes encoding proteins that had direct physical interactions with TBEV virion, TBEV proteins or TBEV RNA; Up- or down-regulated, Genes encoding mRNAs (or proteins) that were up- or down-regulated in response to TBEV infection

^b^For genes from the *cases_only* set the number of loci occupied by harmful variants that are not common in non-Finnish Europeans is given. For genes from the *associated* set the number of variants of any types associated with severe forms of TBE at significance level of 0.01 is given

In most cases (17 genes out of 19) we found that harmful alleles occupied single polymorphic locus within gene. In the other two cases genes contained three and two such polymorphic loci: (1) for *TLR3* we identified rare harmful alleles in three nonsynonymous SNPs located in the 4th exon (rs35311343, rs201222071, and rs781701275) and (2) for *LTF* two nonsynonymous SNPs (rs61739313, rs377642209) were found.

As described above, using significance level of 0.01, we found 667 genes harboring any genetic variants associated with severe forms of TBE (*associated* set). According to TBEVHostDB, only three out of these 667 genes (*TJP2, ATF6,* and *ARID1B*) were previously mentioned in scientific publications in context of TBEV infection. These three genes were: (1) *TJP2,* containing nonsynonymous SNP rs2309428 in exon 9; (2) *ATF6,* containing synonymous nucleotide substitution rs9482 in exon 16; (3) *ARID1B,* containing intronic SNP (rs372586664). Concerning these three genes, the following information is available in the TBEVHostDB [46]. *TJP2* encodes tight junction protein 2 that may interact with the TBEV protein NS5 [70]. *ATF6* encodes activating transcription factor 6, which is activated in Vero E6 cells infected with TBEV [77]. *ARID1B* contains SNP (rs287886) in the first intron, known to be associated with IL-10 concentration in cerebrospinal fluid of Polish patients with TBE [45].

#### Functional annotation of genes harboring harmful variants in exomes of TBE patients, that are not common in non-Finnish European populations

To found specific biological processes and pathways that might be associated with genes, identified in our study, we applied the DAVID tool. First, we performed functional annotation of genes from the c*ases_only* set (2407 genes). According to DAVID tool, the *cases_only* set of genes was enriched (FDR < 0.05) with genes located in plasma membrane and at the cell periphery and was enriched with genes involved in extracellular matrix (ECM) proteoglycans pathway (Table 4).

**Table 4:** GO terms and biological pathways that were overrepresented (FDR value < 0.05) for the *cases_only* set

Term (GO category or Pathway)	GO class or pathway database	Number of genes annotated by the term	Fold enrichment	FDR value
plasma membrane part	cellular compartments	405	1.28	3.75E-05
cell periphery	cellular compartments	734	1.16	4.92E-04
plasma membrane	cellular compartments	718	1.16	6.42E-04
integral component of plasma membrane	cellular compartments	253	1.29	1.72E-02
ECM proteoglycans	REACTOME pathway	24	2.57	3.66E-02

**Fig. 3:**
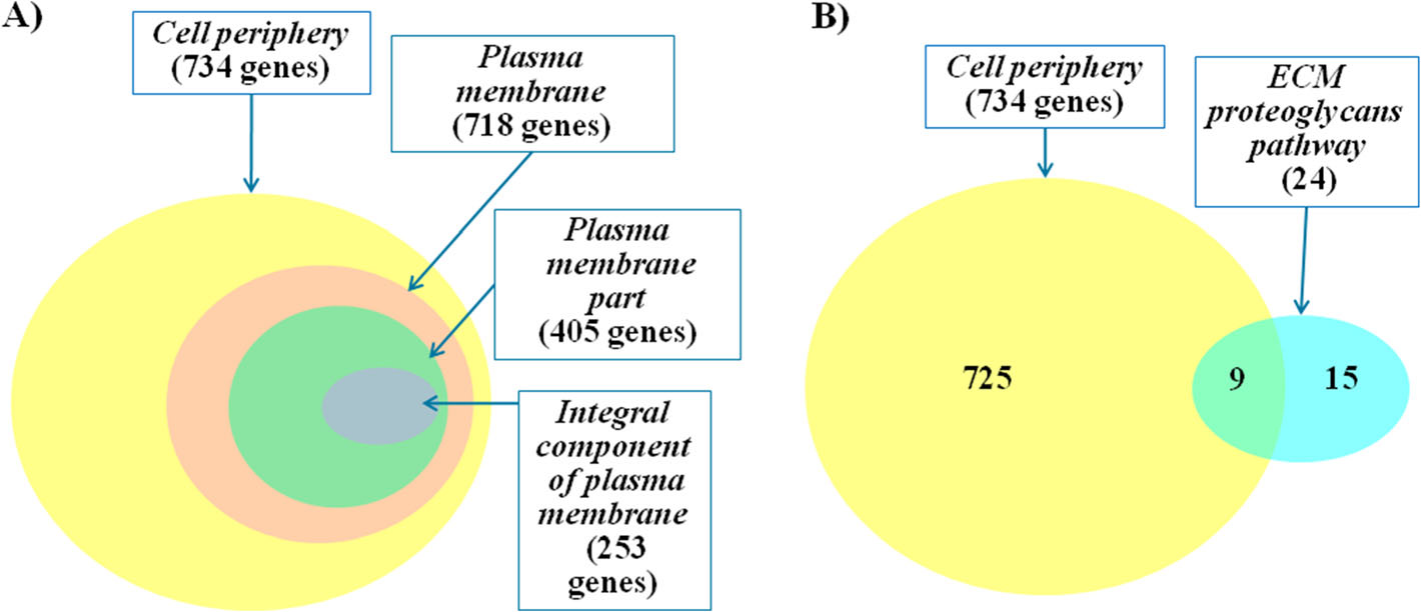
Venn diagram representing the numbers of genes from the *cases_only set,* annotated by overrepresented terms. Panel **a** – numbers of genes annotated by overrepresented GO terms; Panel **b** - numbers of genes annotated by the GO category *Cell periphery* and genes that according to REACTOME pathway database are related to *ECM proteoglycans*

We found that the sets of genes annotated by overrepresented terms have a large portion of the common genes (Fig. 3a). According to Gene Ontology database the term *cell periphery* is a parent GO category and *plasma membrane, plasma membrane part, integral component of plasma membrane* are child terms. Thus, all genes annotated by these three child terms were also annotated by the term *cell periphery*. In addition, 9 out of 24 genes involved in ECM proteoglycans pathway were annotated by *cell periphery* term (Fig. 3b). It should be noted that the other proteins from the ECM proteoglycans pathway might be located in the extracellular space near the cellular membrane [82].

#### Functional annotation of genes associated with severe forms of TBE

**Fig. 4:**
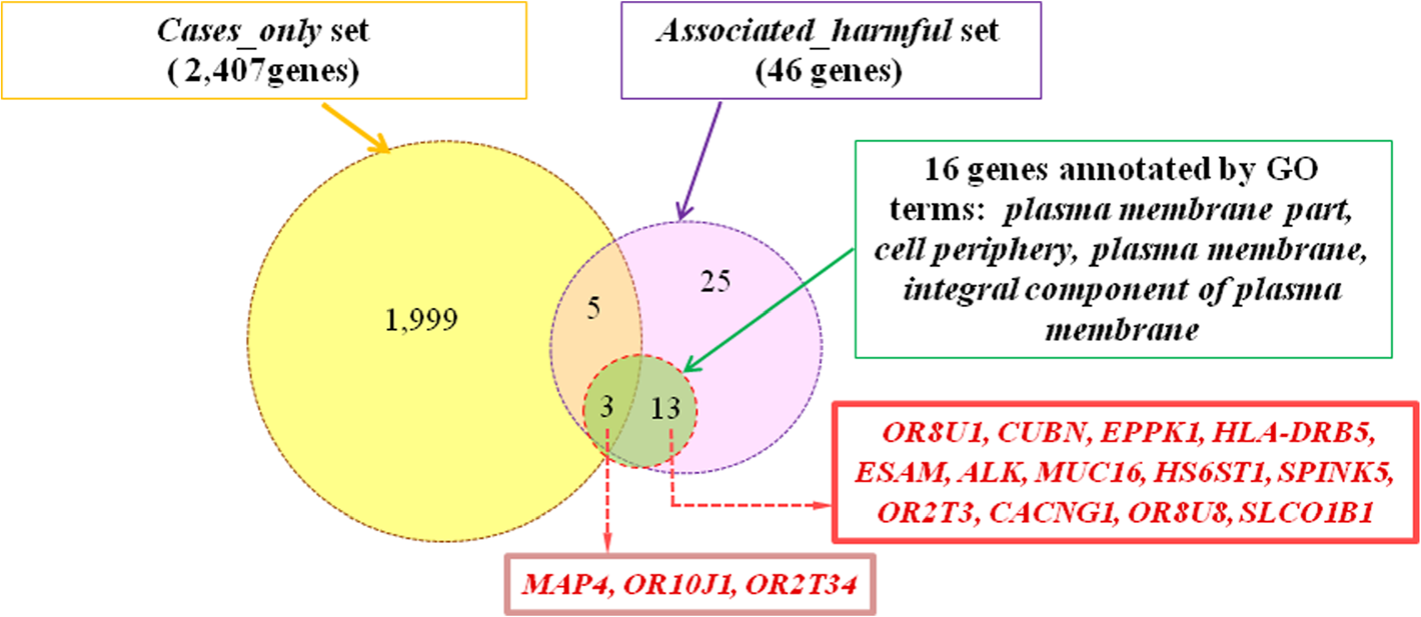
Venn diagram showing the number of genes from the associated_harmful set, that are annotated by GO terms *plasma membrane part, cell periphery, plasma membrane, integral component of plasma membrane* and their intersection with the *cases_only* set

Our attempt to detect significantly overrepresented GO terms or pathways was not successful. We inspected all three lists of genes (Additional file 1: Table S4): (1) *associated* set, containing 667 genes harboring any genetic variants detected at the significance level of 0.01; (2) *associated_possibly damaging* set, including 132 genes; (3) *associated_harmful* set, including 46 genes. In all cases, DAVID tool could not find any significantly enriched GO term, biological pathway or model for biological process. Nevertheless, we hypothesized that the *associated_harmful* set may contain some genes that control the same biological processes or pathways as genes from *cases_only* set. To check this hypothesis we looked at the results of the functional annotation of the genes from the *associated_harmful* set, performed by DAVID tool. We found that *associated_harmful* set contained 16 genes that were annotated by at least one of the four GO terms that were enriched for *cases_only* set at FDR level of 0.05 (*plasma membrane part, cell periphery, plasma membrane, integral component of plasma membrane): MAP4, OR10J1, OR2T34, OR8U1, CUBN, EPPK1, HLA-DRB5, ESAM, ALK, MUC16, HS6ST1, SPINK5, OR2T3, CACNG1, OR8U8, SLCO1B1* (Fig. 4). Three of these genes (*MAP4, OR10J1,* and *OR2T34*) were found in the fraction of genes shared by cases_only and associated_harmful sets. Thus we found that the *associated_harmful* set contained approximately equal (or even slightly larger) fraction of genes (34.5% or 16 genes out of 46), annotated with at least one of the four GO terms named above, compared to *cases-only set* (30.5% or 734 out of 2407).

#### Identification of PPIs between proteins, located at the cell periphery and from the ECM proteoglycans pathway, and proteins, described in the TBEVHostDB

As noted above, all genes from the *cases_only* set, annotated by overrepresented terms (GO categories or pathway names) (Table 4) can be located near the membrane and therefore of them may be functionally related (being involved in the same signaling pathway). The total number of such genes was 749 (Additional file 1: Table S6). Based on this observation we assumed that these 749 genes (designated below as *ECM proteoglycans - cell periphery* set) may constitute the core of the network involved in the response to TBEV infection.

To test this hypothesis, we set the task of building a PPI network, involving genes/proteins from the *ECM proteoglycans - cell peripher*y set and genes/proteins from the TBEVHostDB, comprising genes related to the host response to TBEV infection [46].

Using GeneMANIA Cytoscape plugin [67], we reconstructed PPI network, involving genes/proteins from the *ECM proteoglycans - cell periphery* set and genes/proteins from the TBEVHostDB. Initially, these PPIs involved 430 connected components (data not shown). Using Cytoscape, we identified 154 genes/proteins from the *ECM proteoglycans - cell periphery* set that had physical interactions with genes/proteins from the TBEVHostDB. In total, 99 genes from the TBEVHostDB were involved in these PPIs. (Additional file 1: Table S7).

**Fig. 5:**
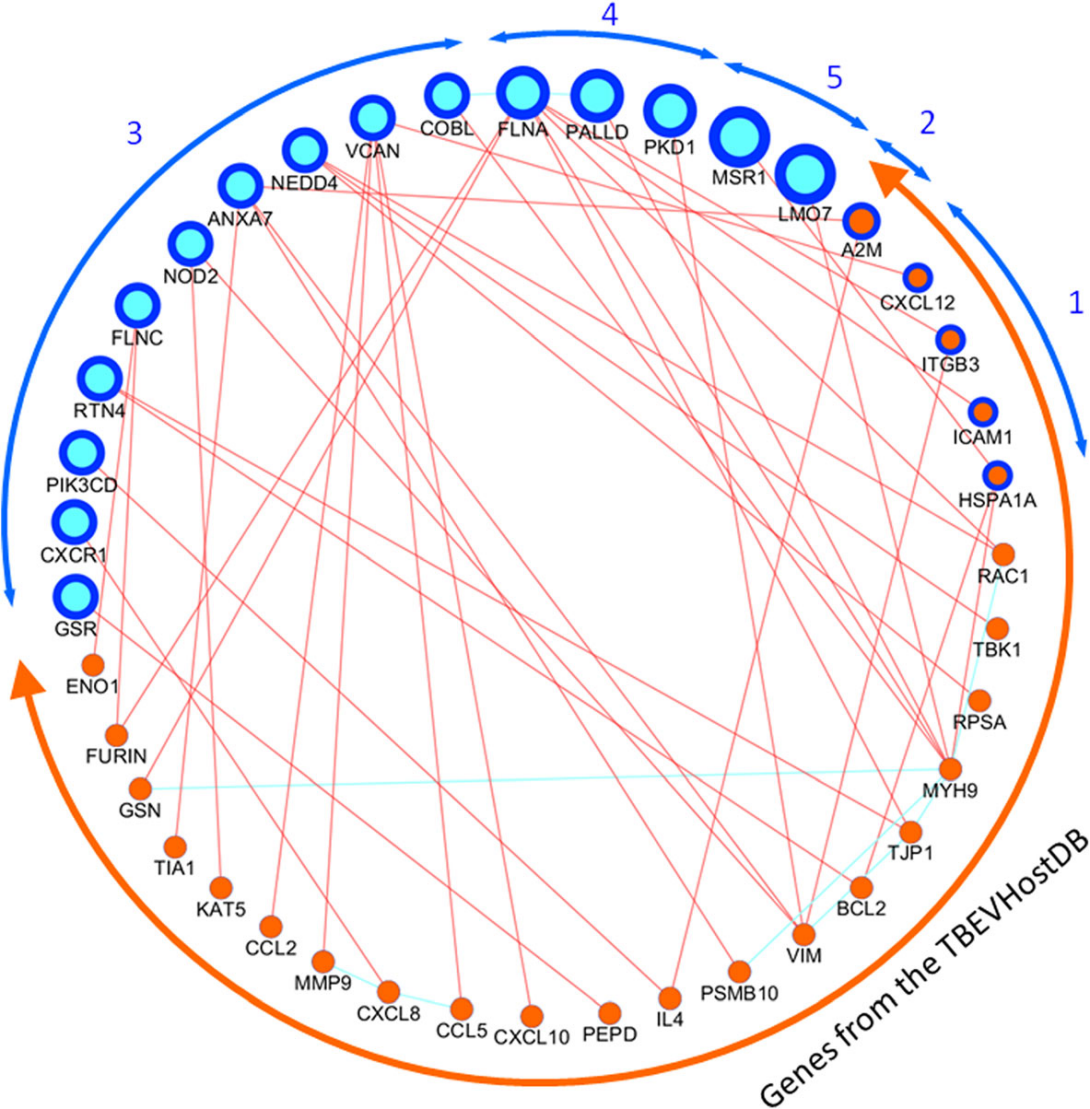
Protein-protein interactions between genes/proteins from the *ECM proteoglycans - cell periphery* set (blue) and genes/proteins from the TBEVHostDB (orange). Genes/proteins, for which the value of N was determined, are denoted by blue border. The size of the objects is proportional to the index N, reflecting the occurrence of the minor alleles harbored by certain gene in exomes of TBE patients (numbers in blue color)

The genes/proteins in the network were ranked according the value of index N, reflecting the occurrence of the minor alleles harbored by certain gene in exomes of TBE patients (22 exomes) (Additional file 1: Table S2). We found that two genes *MSR1* and *LMO7* had the maximal value of index N that was equal to 5 (Fig. 5). Three (*FLNA, PALLD,* and *PKD1*) and ten (*VCAN, ANXA7, NEDD4, FLNC, NOD2, RTN4, COBL, CXCR1, GSR,* and *PIK3CD*) genes had the N values equal to four and three (respectively). For the other 27 and 110 genes (these genes are not presented in the Fig. 5) the values of the N index were equal to two and one. The list of 154 genes from the network and their N indexes are presented in the Additional file 1: Table S8. The references to scientific publications, describing protein-protein interactions, involving 15 genes that were mentioned above (*N* > 3), are presented in Additional file 1: Table S9.

#### Identification of PPIs between genes/proteins from the *associated_harmful* set and genes/proteins from the TBEVHostDB

**Fig. 6:**
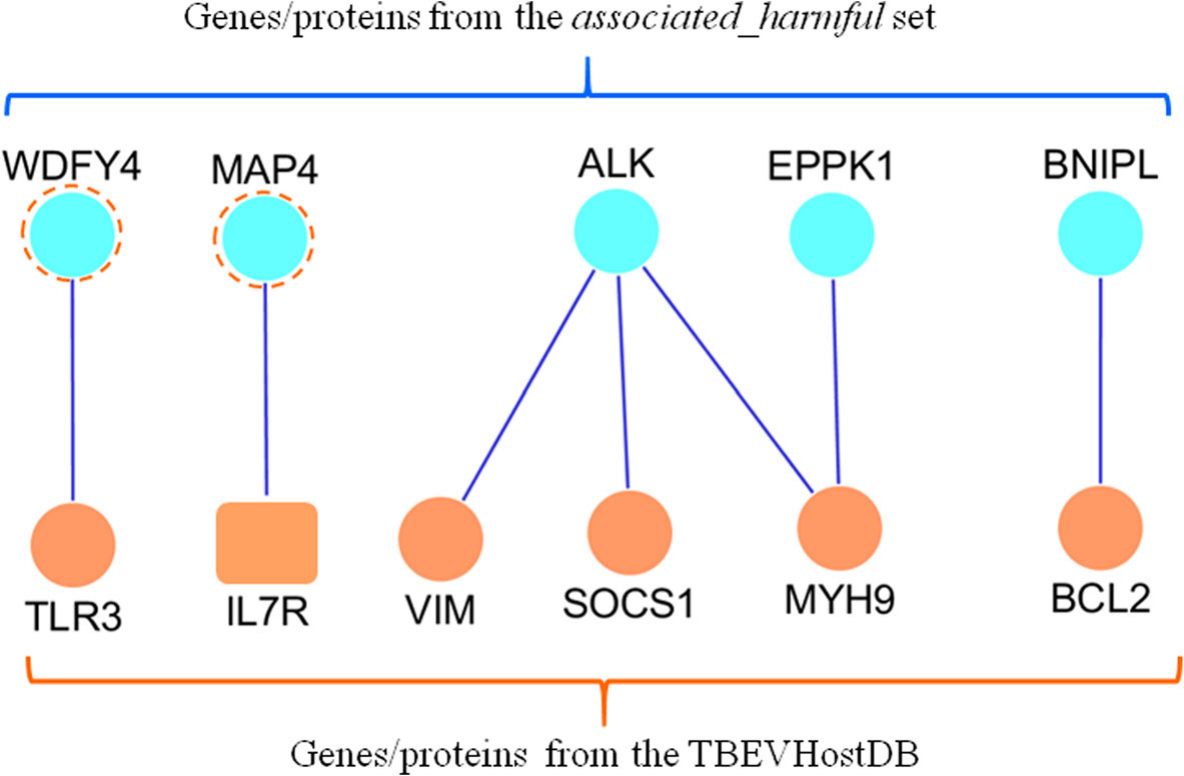
Physical interactions between genes/proteins from the *associated_harmful* set and genes/proteins from the TBEVHostDB extracted from the GeneMANIA database and checked manually. Circles denote proteins; rounded rectangle denotes mRNA. Dashed circles denote two genes/proteins that were also found in *cases_only* set

Using GeneMANIA [67] and Cytoscape [83], we reconstructed a PPI network involving genes/proteins from the *associated_harmful* set and genes/proteins from the TBEVHostDB. This network involved 98 connected nodes (data not shown). A total of 93 out of 98 nodes denoted genes from *TBEVHostDB* and the rest 5 nodes denoted genes from the *associated_harmful* set. According to GeneMANIA, the later five genes/proteins (*WDFY4, MAP4, ALK, EPPK1,* and *BNIPL*) had physical interactions with seven genes/proteins from the TBEVHostDB (Fig. 6). The reliability of these interactions was checked manually by reviewing the literature. According to the scientific publications describing these interactions (Additional file 1: Table S10), six physical interactions involved proteins (PPIs) and the seventh interaction involved IL7R mRNA and MAP4 protein [84]. Two of these five genes genes/proteins (*WDFY4* and *MAP4*) were also found in the *cases_only* set. Three genes (*MAP4, ALK, EPPK1)* were identified previously among 16 genes from the *associated_harmful* set that are annotated with at least one of four closely related GO terms (*plasma membrane part, cell periphery, plasma membrane, integral component of plasma membrane,* Fig. 4). This finding confirms the hypothesis that the disturbances of genes, encoding components of the cell periphery, can be related to predisposition to severe forms of TBE.

## Discussion

### Whole-exome sequencing of Russian patients with severe forms of TBE

It is known that predisposition to severe forms of diseases, in particular, viral diseases is a multifactorial trait, controlled by the both environment and genetic factors [27, 28, 85–87]. The identification of genes predetermining susceptibility to TBEV infection is an ongoing challenge for modern molecular and medical genetics.

Ticks that carry TBEV may be found in a limited area (only in Central Europe, Baltic and Scandinavian countries, and the Russian Federation) [3, 4, 88], largely because of this the number of studies aimed at identifying the genetic factors controlling the host response to TBEV infection is not numerous. In a previous study we sequenced the exomes only from six TBE patients and seven individuals from the control cohort, and subsequently, utilizing a candidate gene approach, identified *MMP9* as a new gene, associated with predisposition to TBE in a Russian population [40]. In the current report we present an exome-wide search of genes associated with severe forms of tick-borne encephalitis based on exome sequencing of DNA from blood samples of 22 Russians patients and 17 individuals from the control cohort.

Relying on data from ExAC and ANOVAR databases, we identified possibly harmful genetic variants in the exomes of TBE patients and in the exomes of the control group that were not common in non-Finnish Europeans (MAF < 0.05). On this basis, we identified 2407 genes that harbored such harmful variants only in exomes of TBE patients and did not contain any rare harmful variants in exomes of the control individuals (Fig. 1 and Additional file 1: Table S2).

Due to limited number of blood samples (22 from TBEV infected patients and 17 from the control cohort) we could not identify SNPs associated with severe forms of TBE infection with *P* values satisfying array-wide level of significance (< 3 * 10^− 7^). Using suggestive significance level (*P* < 0.01) we identified 1100 polymorphic loci (Additional file 1: Table S3) and selected 667 genes (*Associated* set) (Additional file 1: Table S4) that harbored these 1100 polymorphic loci (Table 1, Fig. 2). According to ANNOVAR database, 169 of these 1100 polymorphic loci were possibly or probably damaging (Fig. 2,) and these 169 loci were located within the bodies of 46 genes (*Associated_harmful* set (Table 1 and Additional file 1: Table S4)).

Thus, none of the variants identified in the current study reached the genome-wide significance. Due to the unrealistic effect size for complex diseases needed to achieve statistical significance with the given sample size we employed additional methods to analyze our unique dataset. We came to a decision not to exclude the whole bulk of variants from our consideration and to carry out the functional annotation and analysis of PPIs for (1) genes, harboring rare harmful variants in TBE patients only (*cases_only* set, Additional file 1: Table S2) and (2) genes, harboring variants, associated with severe forms of TBE at *P*-value less than 0.01 (*associated*, *associated_possibly damaging* and *associated_harmful* sets, Additional file 1: Table S4). Our belief that variants, that were revealed at suggestive significance level (P < 0.01), may be informative, was based on the following considerations. Genome- and exome-wide association (GWA and EWA) studies are widely used to identify genetic factors associated with common diseases and traits [48]. However, these studies are puzzling because they appeared to explain only a small proportion of the heritability of the complex traits [47, 89]. For example, according to [90], 97 loci identified at the genome-wide significance accounted for only 2.7% of the body mass index variation. At the same time, polygene analyses suggested that SNPs with *P* values well below genome-wide significance added significantly to the phenotypic variance explained. Thus, the problem of “missing heritability” of complex traits is broadly discussed [47, 91]. An idea that missing heritability could be due to genetic interactions, if the disease involves interactions within or among pathways was also proposed [47, 92]. On the other hand the approach based on integrating the association signal from GWAS data sets into the human PPI network and subsequent functional analysis of genes harboring variants that do not reach genome-wide significance yielded promising results [62, 93, 94].

In view of the above, we applied the a system biology approach to the whole sets of genes: (1) harboring rare harmful variants identified in patients with TBE; and (2) containing variants associated with severe forms of the disease with low *p* value (0.01). The approach was based on combining of the functional enrichment analysis with PPI network reconstruction and analysis.

### Genes, found in the intersection between the cases-only set and associated_harmful set

Using two different approaches to identify genes that may potentially affect the course of the disease, we formed several lists of candidate genes (*cases_only* set*, associated* set*, associated_possibly damaging* set, and associated_harmful set) (Table 1). We found 26 genes in the intersection of the *cases_only* set and *associated_possibly_damaging* (132 genes) set. Eight of these 26 genes were shared between *cases-only* set and *associated _harmful* set (Table 2). That meant that each of these eight genes satisfied the following conditions: (1) the gene harbored at least one rare harmful variant in exomes of TBE patients but did not contain any such variant in exomes of the control individuals; (2) the gene contained at least one harmful variant, associated with severe forms of TBE at P value less than 0.01. In our opinion, these eight genes are of the greatest interest for further study. None of these genes has been studied previously in animal models in the context of susceptibility or resistance to TBEV infection. However, as described above, two genes (*MAP4* and *WDFY4)* had physical interactions with genes/proteins from the TBEVHostDB (Fig. 6 and Additional file 1: Table S10). For all these eight genes, we were able to find data, suggesting a potential mechanism of their acting on the TBE infection.

*MAP4* encodes microtubule associated protein 4. Microtubules are involved in the trafficking of internalized virus from early endosomes to lysosomes for uncoating [95]. MAP4 was found to be one of the human proteins, exploited by HIV in order to replicate, and bearing strong signatures of positive selection [96]. Knockdown of MAP4 produces post-fusion and pre-nuclear translocation impairment in HIV-1 replication [97]. Thus, mutations in *MAP4* gene may alter viral trafficking and, consequently, affect the infectivity of TBEV. The hypothesis that MAP4 can be a risk factor determining severity of infection is supported by the fact that MAP4 can interact with IL7R mRNA [84] (Fig. 6, and Additional file 1: Table S10). According to [76], IL7R (also known as CD127) is involved in response to TBEV infection: in patients with TBE the expression of this protein was decreased in activated (CD38+, Ki67+) CD8 T cells at the peak of their expansion at day 7 after hospitalization.

*WDFY4* (WDFY family member 4) encodes WD repeat- and FYVE domain-containing protein 4. Recently it was reported that this gene contains variants, associated with systemic lupus erythematosus [98] and clinically amyopathic dermatomyositis [99]. It was also found that WDFY4 protein interacted with pattern recognition receptors such as TLR3, TLR4, TLR9 (that play a key role in the innate immune system) and MDA5 (which activate antiviral signaling), and augmented the NF-κB activation by these receptors [99]. *TLR3*, encoding one of these pattern recognition receptors, was annotated in TBEVHostDB: *TLR3* possesses allelic variant associated with severity of TBEV infection in the Russian and the Lithuanian populations [38, 44]. We assume that harmful nonsynonymous substitutions in *WDFY4* can also influence the course of TBE infection.

*ACTRT2* encodes actin-related protein T2 (also known as ARPM2). Similar to other cytoplasmic actin-related protein subfamily members, this protein may be involved in cytoskeletal organization [100]. It is known that TBEV and other flaviviruses use the cytoskeleton network of the cell as a transport system and as a guide to the site of virus replication in the cell [101–103]. Thus, disruption of the actin related protein T2 may affect the intracellular trafficking of TBEV, influencing the course of the disease.

*KLHL25* encodes kelch-like protein 25. This protein is a component of the BCR (KLHL25) E3 ubiquitin ligase complex, which targets and cleaves translation repressor protein 4E-BP1 [104]. 4E-BP1 inhibits cap-dependent translation by binding to the translation initiation factor eIF4E. Flaviviruses use several mechanisms to facilitate translational competence, including activation of the cap-dependent machinery [105, 106]. Thus, pathogenic variants identified in *KLHL25* gene can disrupt the activity of the E3 ubiquitin ligase complex that cleaves 4E-BP1, impairing translational control in the host cell and promoting more effective translation of viral proteins.

*MAP2K3* encodes dual specificity mitogen-activated protein kinase kinase 3 (also known as MKK3, MAPKK3 or MEK3) that belongs to the MAP kinase kinase family. It is involved in inflammatory responses and cellular stress during viral infections [107, 108]. MAP-kinase regulated cytosolic phospholipase A2 activity is essential for production of infectious hepatitis C virus particles [109]. MAP-kinase signaling is involved in inflammatory responses and hepatic cell apoptosis which occur during dengue virus infection [110]. Thus, impaired activity of MAP2K3 protein that may be caused by nonsynonymous mutations can affect signaling pathways controlling antiviral response.

*MBD1* encodes methyl-CpG binding domain protein 1. MBD1 is a transcriptional repressor that binds CpG islands in promoters where the DNA is methylated at position 5 of cytosine within CpG dinucleotides [111]. On the other hand, the antiviral response of the host cell involves transcriptional activation of interferon-inducible genes [28, 112]. Thus, impaired activity of MBD1 may alter antiviral response. For example, the proteins of MBD family (MeCP2, MBD1, MBD2 and MBD4) are involved in the pathogenesis of chronic hepatitis B. The MBD1 mRNA level was up-regulated in patients with chronic hepatitis B compared with healthy [113].

*OR10J1* and *OR2T34* encode two odorant receptors: (1) olfactory receptor family 10 subfamily J member 1, and (2) olfactory receptor family 2 subfamily T member 34. It is known that flaviviruses invade the central nervous system by axonal retrograde transport from the periphery (skin bite), by crossing blood-choroid-plexus barrier or through olfactory bulb neurons [114, 115]. Thus, these two olfactory receptors may be involved in the process of TBEV penetration via the olfactory bulb. It may be interesting to study their roles in this process. In addition, it is known that some olfactory receptors are widely expressed throughout the body and are functional beyond the nasal cavity - with their roles including cytokinesis, muscle regeneration; cell adhesion and migration, chemotaxis, chemokinesis and odorant-mediated serotonin release [116]. According to the Expression Atlas [117], OR2T34 was found to be expressed in leucocytes, thus, mutated protein may in some way alter immune response. In addition, according to the Expression Atlas, OR10J1 is expressed in testis, and both genes are expressed in developing human brain (choroid plexus, 10-th week post conception). The choroid plexus mediates the production of cerebrospinal fluid, maintaining the extracellular environment, required by the brain to function optimally. It can be assumed, that mutant OR10J1 and OR2T34 proteins, expressed in choroid plexus, may disturb barrier function of choroid-plexus, promoting TBEV penetration into the brain.

### Finding genes with known functions

A small portion of genes identified in our study (19 out of 2407 from the *cases_only* set and 3 genes out of 667 genes from the *associated* set) were found in TBEVHostDB (Table 3). This database was created to compile evidence of the potential involvement of genes in response to TBEV infection, published in research articles. Thus, according to TBEVHostDB, only *TLR3* and *ARID1B* have known SNPs (rs3775291 for *TLR3* and rs287886 for *ARID1B*) associated with disease severity in adults from the Lithuanian population and in Russian individuals from Novosibirsk [38, 44] or with IL-10 concentration in cerebrospinal fluid of the Polish patients with TBE [45] respectively.

The other 18 out of 19 genes from the *cases_only* set identified in our study, and two genes from the *associated* set (*TJP2, ATF6*) have the other types of evidence in TBEVHostDB (Table 3). It should be noted that one of these 18 genes, encoding lactotransferrin (*LTF*) that was up- or down-regulated by TBEV infection [80], contained two loci occupied by harmful rare alleles. We propose to keep in mind all these genes as very probable candidates for testing their role in the predisposition to severe forms of TBE in Russians.

### GO analysis

Using DAVID tool, we identified that the *cases_only* set was enriched with genes from the ECM proteoglycans pathway (Table 4). The number of genes that were found to be involved in ECM proteoglycans pathway was 24, and the fold enrichment was 2.6. These finding is in a good agreement with the other studies, investigating the relevance of the ECM proteoglycans pathway to the manifestation of the disease, caused by flaviviruses. The following is known by now. Transcriptome meta-analysis revealed a dysregulation in extra cellular matrix and cell junction associated gene signatures during dengue virus infection in the human monocytic cell line THP-1 [118]. It was found that it was proteolysis of extracellular matrix proteins with serine protease from mosquito saliva that increased viral attachment to heparan sulfate proteoglycans, thereby augmenting dengue virus infectivity *in vitro* [119]*.* In addition, three proteins from the ECM proteoglycans pathway (integrin subunit beta encoded by *ITGB3,* laminin subunits beta 1 and beta 2 encoded by *LAMB1* and *LAMB2)* can interact physically with the whole TBEV particle or with its proteins [69, 72, 120].

We found out that the *cases_only* set was also enriched in genes, annotated by four closely related GO categories (Fig. 3), which indicate localization of proteins near the plasma membrane (*integral component of plasma membrane, plasma membrane part, plasma membrane, cell periphery*). The percentage of such genes in the *cases_only* set was 30.5% (734 out of 2407). Approximately the same percentage of genes (34.5%), encoding components located at cell periphery, was identified for the *associated_harmful* set (16 genes presented in Fig. 4).

This finding is in good agreement with the known mechanisms of viral infection. In order to infect host cell, TBEV particle must gain entry into host cells. Flaviviruses enter host cells by endocytosis, initiated when the virus particles interact with cell surface receptors [121]. Heparan sulfate-containing proteoglycans can function as an attachment receptor for TBEV [122]. But the existence of the other host cell receptors (like beta 1-chain of human integrin (110 kD), encoded by *LAMB1*, alpha 3-chain of human integrin, encoded by *ITGA3* and laminin receptor, 67 kD, encoded by *RPSA*), that can mediate the entry of TBEV particles, was also proposed [72, 120, 123]. The internalization of viral particles into the cell may involve remodeling of the actin cytoskeleton and activation of EGFR-PI3K signaling pathway [124]. The TBEV protein NS5 can associate with membrane protein scribble and impair interferon-stimulated JAK-STAT signaling [125].

### Candidate genes/proteins, interacting with genes/proteins from the TBEVHostDB

To get additional functional characteristics of candidate genes, we reconstructed and analyzed two PPI networks, involving, on the one hand, candidate genes, identified in this study (*ECM proteoglycans - cell periphery* set and *associated_harmful* set), and, on the other hand, genes from TBEVHostDB.

Expecting the first network, we identified 154 candidate genes/proteins from the *ECM proteoglycans - cell periphery* set that had pairwise interactions with genes/proteins from the TBEVHostDB (Additional file 1: Table S8). Using index N (calculated for each gene from the *cases_only* set as a total number of all rare harmful minor alleles that were found in exomes of patients with TBE), we ranked genes, involved in the network. Thus, in this network we found 15 genes that were loaded with harmful variants most heavily (*N* > 3) (Fig. 5). The functional annotation of these genes, containing the ideas concerning their potential relevance to the host response, is presented in Table 5.

**Table 5:** Functional annotation of candidate genes/proteins from the *ECM proteoglycans - cell periphery* set, interacting with genes/proteins from the TBEVHostDB and loaded with harmful variants most heavily (N > 3)

No	Gene	N ^a^	Number of neighbors^b^	Functional annotation
1.	*MSR1*	5	1	*MSR1* gene encodes macrophage scavenger receptor 1. Macrophage scavenger receptors mediate the **endocytosis** of a diverse group of macromolecules. Expression of *MSR1* promotes alternative activation of murine macrophages following hepatic viral infection [126]. On the other hand, it is known, that Flaviviruses enter host cells through the process of clathrin-mediated **endocytosis** [127, 128]. Thus, MSR1 may interfere with this process.
2.	*LMO7*	5	1	*LMO7* gene encodes LIM domain only protein 7 with three conserved domains: (1) the CH (Calponin Homology) domain, known to confer the **actin**-binding ability on many **actin**-associated proteins; (2) the PDZ domain, a region containing a conserved Gly-Leu-Gly-Phe repeat sequence, may take part in protein targeting and protein complex assembly; (3) LIM (Lin-11-Isl-1-Mec-3) domain, that may regulate transcription or cytoskeleton assembly [129]. Thus LMO7 is known to be a nucleocytoplasmic shuttling protein that possesses both **actin**-binding and transcription regulator activities [130]. There are no evidence confirming involvement of LMO7 in response to viral infection, nevertheless we propose that it may play a role in host response, due to its involvement in cytoskeletal reorganization and transcription regulation processes.
3.	*FLNA*	4	7	*FLNA* gene encodes filamin A (also known as ABP-280). Filamin A is ubiquitous dimeric **actin** cross-linking phosphoprotein, located in peripheral cytoplasm, where it promotes orthogonal branching of **actin** filaments and links **actin** filaments to membrane glycoproteins [131].
4.	*PALLD*	4	1	*PALLD* gene encodes palladin, **actin**-associated protein. Palladin is a cytoskeletal scaffold protein that may promote tumor cell invasion by linking extracellular matrix degradation in **actin**-based pseudopods to cell cytoskeleton [132].
5.	*PKD1*	4	1	PKD1 (polycystic kidney disease 1) gene encodes polycystin 1, a glycoprotein with multiple transmembrane domains and a cytoplasmic C-tail. Polycystin 1 is an integral membrane protein, associated with the cytoskeleton [133] and involved in cell-cell/matrix interactions [134]. Polycystin 1 regulates **actin** cytoskeleton rearrangements and directional cell migration [135].
6.	*COBL*	3	1	*COBL* gene encodes cordon-bleu WH2 repeat protein working as a dynamizer of **actin** assembly [136].
7.	*FLNC*	3	2	*FLNC* gene encodes filamin C that is one of three related filamin proteins involved in remodeling of the **actin** cytoskeleton. Filamin C is a predominantly expressed filamin isoform in striated muscles [137].
8.	*RTN4*	3	2	*RTN4* gene encodes reticulon 4 (also known as NOGO), an important axonal growth inhibitor in the adult and developing central nervous system. Reticulon 4 may act as a molecular linker between microtubules and **actin** cytoskeleton in rat vascular smooth muscle cells [138]. RTN4 has been shown to inhibit migration and cell spreading of neuronal and nonneuronal cell types [139].
9.	*NOD2*	3	2	*NOD2* gene can function as a cytoplasmic viral pattern-recognition receptor by recognizing viral ssRNA genome and then by triggering activation of interferon-regulatory factor 3 (IRF3) and **production of interferon-beta** [140].
10.	*CXCR1*	3	1	*CXCR1* gene encodes C-X-C motif chemokine receptor 1 (also known as a **receptor for interleukin 8**). It was found that infection with dengue virus induces interleukin 8 secretion, which increases endothelial cell permeability; this has been proposed as a mechanism for plasma leakage in dengue hemorrhagic fever [141]. It was also found that while infection with West Nile virus the production of interleukin 8 was induced too [142].
11.	*PIK3CD*	3	1	*PIK3CD* gene encodes phosphatidylinositol-4,5-bisphosphate 3-kinase catalytic subunit delta. Phosphoinositide 3-kinases phosphorylate inositol lipids and are involved in the **immune response**. It was found that when human Caco-2 cells were pre-treated with inhibitor of PI3K pathway, TBEV cell entry was efficiently blocked [101]
12.	*VCAN*	3	5	*VCAN* gene encodes versican core protein. This is a large chondroitin sulfate proteoglycan and is a major component of the extracellular matrix. This protein is involved in cell adhesion, proliferation, migration and angiogenesis and plays a central role in tissue morphogenesis and maintenance [143]. Versican is known as an interferon-stimulated gene contributing to fine control of **innate immunity** [144]. In a mouse model it was shown that versican could mediate **inflammatory response** to viral infection and could generate an extracellular matrix that promoted leukocyte infiltration and adhesion [145]
13.	*ANXA7*	3	4	*ANXA7* gene encodes a membrane binding protein annexin A7 possessing integrin binding activity [146]. As a GTPase, annexin A7 interacts with TIA1 in regulating vascular endothelial cell **autophagy** [147]. The functional importance of autophagy during TBEV replication was studied in human neuroblastoma cells; stimulation of autophagy resulted in significantly increased dose-dependent TBEV production, whereas the inhibition of autophagy showed a profound, dose-dependent decrease of the yield of infectious virus [148].
14.	*GSR*	3	1	*GSR* gene encodes glutathione-disulfide reductase, the central enzyme of cellular **antioxidant defense**. Like other flaviviruses, TBEV may promote oxidative stress in the host cell [74]. As a result, the antioxidant defense system may be significantly perturbed. Thus, it was found that the level of glutathione-disulfide reductase was significantly reduced in the red blood cells isolated from individuals with human immunodeficiency virus infection [149] or, on the contrary, was increased in Huh-7 cell line infected with HCV [150].
15	*NEDD4*	3	3	*NEDD4* (neural precursor cell expressed, developmentally down-regulated 4, E3 ubiquitin protein ligase) gene encodes E3 ubiquitin-protein ligase NEDD4. It plays critical role in the regulation of a number of membrane receptors and **endocytic machinery**. The role of Nedd4-like ubiquitin ligases in virus budding has been established. Nedd4 promotes Japanese encephalitis virus replication by suppressing **autophagy** [151] and may facilitate HIV-1 release [152].

^a^N denotes the number of rare harmful minor alleles that were found in exomes of patients with TBE

^b^The number of genes/proteins from the TBEVHostDB that had PPIs with the gene/protein in question

The maximal values of the index N were found for two genes: (1) *MSR1,* encoding macrophage scavenger receptor 1 and (2) *LMO7,* encoding LIM domain only protein 7. As *MSR1* mediates the endocytosis of a diverse group of macromolecules [127], it may interfere with the pathways of virus entry into the host cell. *LMO7* and the other six genes identified at this step *(FLNA, PALLD, PKD1, COBL, FLNC,* and *RTN4*) encode proteins that function in cooperation with actin (Table 5). Actin is a major component of cytoskeleton. As noted above, cytoskeleton reorganization conducive to permeability increase may be induced by flavivirus infection [124, 153].

The functioning of the next four genes presented in Table 5 (*NOD2, CXCR1, PIK3CD,* and *VCAN*) is related to the immune response [101, 140, 141, 144, 145].

The rest three genes (*ANXA7, GSR,* and *NEDD4*) had different functions, but, in each case, we were able to find evidence, pointing to a potential mechanism of their participation in the host response (Table 5).

Examining the second network, we identified five candidate genes/proteins from the *associated_harmful* set (*WDFY4, MAP4, ALK, EPPK1,* and *BNIPL)* that had pairwise interactions with genes/proteins from the TBEVHostDB database (Fig. 6). The functional annotation of these genes presenting its potential relevance to host response is given in Table 6.

**Table 6:** Functional annotation of c*andidate genes/proteins* from the *associated_harmful* set, interacting with genes/proteins from the TBEVHostDB database

No	Gene	Number of neighbors^a^	Functional annotation
1.	*WDFY4* ^b^	1	*WDFY4* gene encodes WDFY family member 4, which has a predicted BEACH domain and five WD domains at the C-terminal side. WDFY4 may interact with pattern recognition receptors TLR3, TLR4, TLR9 (playing a key role in the innate immune system) and MDA5 (activator of antiviral signaling) and may augment the **NF-κB activation** by these receptors. WDFY4 is expressed in dendritic cells, neutrophils, B cells and macrophages [99].
2.	*ALK*	3	*ALK* gene encodes ALK receptor tyrosine kinase, a transmembrane receptor tyrosine kinase from the insulin receptor superfamily. The growth factors pleiotrophin and neurite growth-promoting factor 2 are ligands for ALK and upon ALK activation, insulin receptor substrate-1 (IRS-1) and other substrates are phosphorylated. ALK can also drive **NF-κB activation**, and that activation is increased with the presence of insulin receptor substrate-1 [154]
3.	*MAP4* ^b^	1	MAP4 gene encodes **microtubule** associated protein 4. **Microtubules** are involved in the trafficking of internalized virus from early endosomes to lysosomes for uncoating [95]. MAP4 is one of the human proteins exploited by HIV in order to replicate and bearing strong signatures of positive selection [96]. Knockdown of *MAP4* produces post-fusion and pre-nuclear translocation impairment in HIV-1 replication [97].
4.	*EPPK1*	*1*	*EPPK1* gene encodes epiplakin 1. Epiplakin 1 is a member of the plakin family with multiple copies of the plakin repeat domain. Epiplakin is associated with assembled **intermediate filaments** over keratin monomers and with vimentin intermediate filaments network [155]. Together with the other components of the cytoskeleton, the intermediate filaments have been observed to be involved in flavivirus entry, assembly, and budding processes in the host cells [156]. For example, Zika virus infection causes a drastic reorganization of microtubules and **intermediate filaments** forming cage-like structures surrounding the viral replication factory [157]
5.	*BNIPL*	*1*	*BNIPL* gene encodes BCL2 interacting protein like. It is a member of BNIPL family, which interacts with **apoptosis** regulators Bcl-2 and Cdc42. BNIPL-2 may be a linker protein located at the front end of Bcl-2 pathway for DNA fragmentation and Cdc42 signaling for morphological changes during **apoptosis** [158]. Direct killing of the neurons by **apoptosis** has been reported for such flaviviruses as Langat virus and TBEV in cell culture [159, 160]. Enforced BCL2 expression may attain Japanese encephalitis virus persistence in baby hamster kidney (BHK-21) and Chinese hamster ovary (CHO) cells by restriction of virus-induced cytopathic effects [161]

^a^The number of genes/proteins from the TBEVHostDB that had PPIs with the gene/protein in question

^b^*WDFY4 and MAP4* are two of eight genes found in the intersection between the *cases-only* set and *associated_harmful* set (Fig. 2)

Two genes *WDFY4* and *ALK* may augment the NF-κB activation [99, 154]. NF-κB is a key regulator of immune and inflammatory processes, which can induce the occurrence of inflammatory reaction and further affect its development, such as the unlimited proliferation of inflammatory cells, cell immortalization, vascular tissue invasion and metastasis [162]. It was found that NF-κB plays an important role in the occurrence and development of viral encephalitis. Patients with TBE had increased concentration of NF-κB in cerebrospinal fluid, than individuals from the control group [163]. Moreover, NF-κB level in serum and cerebrospinal fluid of children with viral encephalitis was positively related to the severity of the disease. [164].

The functioning of the other two genes (*MAP4* and *EPPK1)* is related to intermediate filaments and microtubules, which are components of cytoskeleton [97, 155]. Reorganizations of the cytoskeleton of the host cells come along the Flaviviridae life cycle - its entry, assembly, and budding processes [156].

The fifth gene is *BNIPL,* involved in the regulation of apoptosis. Apoptosis is one of the mechanisms of the cytopathic effect of the TBEV. TBEV can cause programmed cell death both *in vitro* and *in vivo* [165].

### The limitations of the study: human genetics is not the sole determinant of disease severity

Identification of genes and biological pathways associated with severe forms of TBE is an important challenge of modern genetics. For this purpose, we performed an exome-wide association study (EWAS) in Russian patients with severe forms of TBE. Limitations of the GWAS and EWAS approaches have been widely discussed [166–168].

It is also worth keeping in mind that human genetic factors are not the only ones determining the severity of the disease. A number of host characteristics may contribute to the disease progression, such as: (1) advanced age [11, 12, 169]; (2) male gender [169]; (3) decreased immune status [11, 170, 171]; (4) previous vaccinations and vaccine types [22, 172].

Differences in severity of TBE may also be due to characteristics of individual TBEV subtypes. As mentioned earlier, three genetically distinct subtypes of viruses cause TBE (Far-Eastern subtype, Siberian subtype and European subtype) and each of these subtypes causes clinically distinct diseases with varying degrees of severity [12, 14, 173]. Experiments on animals supported the idea that different TBEV subtypes possess different pathogenic activities [174, 175].

Not only the TBEV subtype determines the severity of the disease. The relationship between the structure of the TBEV strains and their virulence or pathogenic properties had been shown for all three TBEV subtypes in both humans and mice [78, 173, 176–178]. Therefore, even if we assume that all patients involved in our study were infected with the same TBEV subtype, genetic variations in TBEV could affect the course of the disease.

Studies on genetic variants associated with viral diseases are limited by the lack of proper controls. To search for genetic variants associated with severe forms of infectious diseases, it is recommended to form a control group consisting of individuals with clinically confirmed infection that was asymptomatic or mild [28]. However, in the case of tick-borne encephalitis, it is not easy to fulfill this recommendation. First, people with mild forms of TBE usually do not seek medical care. Secondly, according to the official governmental vaccination program, vast majority of active people in endemic areas of the Russian Federation (including Novosibirsk region) have been vaccinated [179]. Therefore, mild forms of the disease can be due to proper vaccination rather than genetic resistance.

In the current study, DNA samples from individuals of both sexes were analyzed. The ages of patients involved in the study varied considerably (from 15 to 69 years): seven individuals were under the age of 30 years, seven individuals were between the ages of 40 and 60 and eight individuals were between the ages of 65 and 69. Thus, we may assume that age and gender could not predispose to such severe forms of the disease that were observed in 22 patients involved in the study. However, in order to exclude any potential impact of other host-related factors (immune status, etc.) or viral genetic factors (subtypes and strains), further studies are needed.

## Conclusion

The main goal of our study was to find genes, associated with severe forms of TBE, and to identify biological pathways, through which pathogenic genetic variants may influence the severity of the disease. For this purpose, we performed exome-wide search for genes responsible for severe forms of TBE in 39 DNA samples from Russian individuals. Combining several theoretical approaches (identification potentially pathogenic variants, identification of the variants, that were not common in non-Finnish Europeans, and identification of genetic variants, associated with severe forms of TBE), we detected eight new candidate genes (*MAP4, WDFY4, ACTRT2, KLHL25, MAP2K3, MBD1, OR10J1,* and *OR2T34)* that may be the most probable candidates affecting the cause of TBEV infection in the Russian population.

Functional annotation of genes harboring rare harmful variants found in exomes of TBE patients or genes harboring variants associated with TBE indicated that the components of ECM proteoglycans pathway and the components of the cell periphery with high probability can be modulators of the course of the TBE disease.

Further, expecting PPI networks, involving candidate genes and genes from TBEVHostDB database, we identified hubs, that is, candidate genes that had the greatest number of physical associations with genes from the TBEVHostDB. Among them were (1) 15 genes harboring the maximal number of the rare pathogenic variants in exomes of TBE patients (*MSR1, LMO7, FLNA, PALLD, PKD1, VCAN, ANXA7, NEDD4, FLNC, NOD2, RTN4, COBL, CXCR1, GSR,* and *PIK3CD*), and (2) five genes harboring genetic variants, associated with severe forms of TBE (*WDFY4, ALK, MAP4, EPPK1,* and *BNIPL*). Further studies of these genes on larger sets of samples from TBE patients are required for verification the roles of candidate genes in host-virus interaction during TBEV infection. The identified genes and genetic variants also need to be further verified experimentally in model systems (cells or animals) and the work on the collection of exome data from other TBE patients should be continued.

## Additional files

Additional file 1: **Table S1.** Genes harboring rare pathogenic variants revealed in exomes of patients with TBE (*cases* set) and in exomes of the control individuals (*control* set). **Table S2.** *Cases_only* set, containing genes that were unique for the *cases* set. **Table S3.** Genetic variants (SNPs or Indels) associated with severe forms of TBE at *P*-value less than 0.01. **Table S4.** Three sets of genes harboring genetic variants associated with severe forms of TBE at P-value less than 0.01: (1) *associated* set; (2) *associated_possibly damaging* set; (3) *associated_harmful* set. **Table S5.** Genetic variants found in eighty genes shared by the *cases_only* (2407 genes) set and the *associated_possibly damaging* set (132 genes). **Table S6.** *ECM proteoglycans - cell periphery* set, containing genes from the *cases_only* set, annotated by overrepresented terms. **Table S7.** PPIs between genes/proteins from the *ECM proteoglycans - cell periphery* set and genes/proteins from the TBEVHostDB. **Table S8.** The list of 154 genes/proteins from the *ECM proteoglycans - cell periphery* set, that were involved in PPIs network with genes/proteins from TBEVHostDB and their N indexes. **Table S9.** PPI interactions involving 15 top genes/proteins from the *ECM proteoglycans - cell periphery* set with *N* > 3. References were checked manually by reviewing the literature. **Table S10.** PPI interactions involving 5 genes/proteins from the *associated_harmful* set. References were checked manually by reviewing the literature. (DOCX 13 kb)

## Abbreviations

EWAS: Exome-wide association studies
GWAS: Genome-wide association studies
MAF: Minor allele frequency
NFE: Non-Finnish European
PIs: Pairwise interactions
PPIs: Protein-protein interactions
SNPs: Single nucleotide polymorphisms
SNVs: Single-nucleotide variants
TBE: Tick-borne encephalitis
TBEV: Tick-borne encephalitis virus
