# Supplementary material for: Exome-wide search and functional annotation of genes associated in patients with severe tick-borne encephalitis in a Russian population
Source: BMC Med Genomics. 2019 May 24;12(Suppl 3):61. doi: 10.1186/s12920-019-0503-x (PMC6533173; doi:10.1186/s12920-019-0503-x)
Supplement: Supplementary file 1 — Table S1. Genes harboring rare pathogenic variants revealed in exomes of patients with TBE (cases set) and in exomes of the control individuals (control set). Table S2. Cases_only set, containing genes that were unique for the cases set. Table S3. Genetic variants (SNPs or Indels) associated with severe forms of TBE at P-value less than 0.01. Table S4. Three sets of genes harboring genetic variants associated with severe forms of TBE at P-value less than 0.01: (1) associated set; (2) associated_possibly damaging set; (3) associated_harmful set. Table S5. Genetic variants found in eighty genes shared by the cases_only (2407 genes) set and the associated_possibly damaging set (132 genes). Table S6. ECM proteoglycans - cell periphery set, containing genes from the cases_only set, annotated by overrepresented terms. Table S7. PPIs between genes/proteins from the ECM proteoglycans - cell periphery set and genes/proteins from the TBEVHostDB. Table S8. The list of 154 genes/proteins from the ECM proteoglycans - cell periphery set, that were involved in PPIs network with genes/proteins from TBEVHostDB and their N indexes. Table S9. PPI interactions involving 15 top genes/proteins from the ECM proteoglycans - cell periphery set with N > 3. References were checked manually by reviewing the literature. Table S10. PPI interactions involving 5 genes/proteins from the associated_harmful set. References were checked manually by reviewing the literature. (DOCX 13 kb) [file 12920_2019_503_MOESM1_ESM.docx]

**About this supplement**

This article has been published as part of *BMC Medical Genomics Volume 12 Supplement 2, 2019: Selected articles from BGRS\SB-2018: medical genomics (part 2).* The full contents of the supplement are available online at https://bmcmedgenomics.biomedcentral.com/articles/supplements/volume-12-supplement-3.
